# Supplementary material for: TrpV1 receptor activation rescues neuronal function and network gamma oscillations from Aβ-induced impairment in mouse hippocampus in vitro
Source: eLife. 2018 Nov 12;7:e37703. doi: 10.7554/eLife.37703 (PMC6281315; doi:10.7554/eLife.37703)
Supplement: Source code 1. [file elife-37703-code1.docx]

% SPIKE PHASE-COUPLING ANALYSIS

%This script has been made to relate the PC spiking activity to ongoing gamma oscillations. Action Potentials (AP) are detected by setting the amplitude threshold and the instantaneous phase of gamma oscillation is calculated using Hilbert transform in order to determine the phase-angle at which each AP occurred during ongoing oscillations. Field potential recording signal was pre-processed in Clampfit using a band pass filter set to 20-60Hz (high-pass: RC-single pole, low-pass: Gaussian.

% Name "AP" the variable containing the action potentials signal.

% Name "LFP" the variable containing the field potential recording signal.

clc, clf;

% PHASE-COUPLING

mVrange=linspace(min(AP),max(AP));

threshold=mVrange(60); % Amplitude threshold value can be change in order to detect all APs.

[~,indAP]=findpeaks(AP,'MinPeakHeight',threshold); hilLFP=hilbert(LFP); angLFP=angle(hilLFP); angLFP=angLFP-2*pi*floor(angLFP/(2*pi));

disp('AP phase-angles:') posLFP=angLFP(indAP) % AP phase-angles rose(posLFP) %AP phase-angles Polar plot

% 0 correspond to 0pi

% 180 correspond to +/-pi

% RAYLEIGH'S Z-TEST

TotalAP=length(indAP);

u = sum(exp(1i*posLFP))/TotalAP; VecLength=abs(u);

r=VecLength; n=TotalAP; R=r*n; z=R^2/n;

disp('Rayleigh test p-value:')

pval=exp(sqrt(1+4*n+4*(n^2-R^2))-(1+2*n)) %Rayleigh test p-value shg
